# Deciphering splenic marginal zone lymphoma pathogenesis: the proposed role of microRNA

**DOI:** 10.18632/oncotarget.25487

**Published:** 2018-07-06

**Authors:** Jacob E. Robinson, Christine E. Cutucache

**Affiliations:** ^1^ Deptartment of Biology, University of Nebraska at Omaha, Omaha, NE 68182, USA

**Keywords:** splenic marginal zone lymphoma, microRNA, CAV1, caveolin-1, 7q

## Abstract

Splenic marginal zone lymphoma (SMZL) is a malignancy of mature B-cells that primarily involves the spleen, but can affect peripheral organs as well. Even though SMZL is overall considered an indolent malignancy, the majority of cases will eventually progress to be more aggressive. In recent years, the gene expression profile of SMZL has been characterized in an effort to identify: 1) the etiology of SMZL, 2) biological consequences of SMZL, and 3) putative therapeutic targets. However, due to the vast heterogeneity of the malignancy, no conclusive target(s) have been deciphered. However, the role of miRNA in SMZL, much as it has in chronic lymphocytic leukemia, may serve as a guiding light. As a result, we review the comprehensive expression profiling in SMZL to-date, as well as describe the miRNA (and potential mechanistic roles) that may play a role in SMZL transformation, particularly within the 7q region.

## INTRODUCTION

Splenic marginal zone lymphoma (SMZL) is a low-grade, mature B-cell lymphoma, primarily involving the spleen with variable progression seen in the bone marrow and peripheral blood [1, 2]. SMZL accounts for less than 2% of all lymphoid malignancies and is responsible for less than 1% of non-Hodgkin's lymphoma cases [3–5]. SMZL is considered an indolent B-cell lymphoma as the median overall survival (OS) for SMZL cases is between 8 and 11 years, but clinical presentations remain very heterogeneous [6–8]. Most importantly, it is estimated that 70% of SMZL cases will at some point require treatment for worsening symptoms [2, 9], and approximately 30% of SMZL patients will display a more aggressive prognosis with potential for progression to more lethal lymphomas and a decreased overall survival [1, 10–14]. Due to the limited number of available cases as well as the heterogeneous nature of SMZL, it is very difficult to differentiate indolent and aggressive SMZL cases, resulting in treatment inconsistencies and discrepancies among predicted clinical prognoses. Consequently, further investigations into the biological mechanism(s) that result in SMZL development are essential for improving the diagnostic and prognostic reliability.

### Etiology of SMZL

In an effort to elucidate the biological mechanisms of SMZL, research involving gene expression analyses and chromosomal aberration studies have been conducted. Specifically, SMZL presents with genomic instability in approximately 75% of cases, resulting in one of the highest percentages compared to other B-cell lymphomas, and accentuating the variable nature of the disease [15]. This variability offers potential opportunities in identifying diagnostic and subsequent treatment targets. Thus far, the most common chromosomal abnormality is a 7q deletion occurring in 30% to 40% of patients. The loss of 7q regions is seen much more frequently in SMZL compared with similar B-cell neoplasms, and thus, it has been proposed as a primary diagnostic marker [16–19]. The primary region resulting in the loss of heterozygosity (LOH) has been identified between 7q21 and 7q33, but the precise chromosomal locations responsible, and resulting mechanisms, remain unknown [20–22]. Further, the direct effects of the 7q deletion on SMZL pathogenesis also remains controversial.

In addition to the deletion within the 7q region, there are a plethora of other cytogenetic abnormalities identified in previous studies. These include various, inconsistent translocations, gains primarily in 3q, 4q, 5q, 9q, 11q, 12q, and 20q, and losses occurring in 6q, 8p, 13q, 15q, and 17p [19, 23–27]. The many discoveries of chromosomal abnormalities have assisted in the identification of SMZL, but understanding the mechanistic progression of the disease has still eluded investigators in cytogenetic investigations.

Along with the challenge posed by the heterogeneity of chromosomal aberrations, finding patterns among other biological indicators has proven equally as difficult. While cytogenetic investigations have been cited for their importance, it has been suggested that targeting gene mutations will provide more clinical relevance than the former [28]. In an effort to identify crucial genetic abrogates, a number of genetic mutations and deletions have been investigated for their roles in the NF-κB pathway, cell communication, apoptosis, metabolism, cell cycle control, lymphocyte development, and chromosomal and transcriptional regulation (Table 1).

**Table 1:** Summary of significantly deregulated genes and their resultant affected pathways in SMZL

Pathway	Deregulated genes	Citation
NF-κB	*IKBKB, TNFAIP3* (A20), *BIRC3, TRAF3, MAP3K14, CD40, SYK, BTK, PKCA, REL, TRAF5, PTPRC, PTPN1, TNFRSF5, LTB, MYD88, CARD11, FAS, CREBBP, NFKBIZ, KLF2*	[6, 28–38, 41]
Cell Communication	*MS4A2, SYK, TOSO, SELL, LPXN, PTPRC, PTPN1, RASSF2, BIRC3, TNFRSF5, TRAF3, TRAF5, ENPP2, BTK, PDE4B, PLEXINA2, ARHGAP25, ARHGAP32, MYCBP2, FLNC, LCP1, CALU*	[30, 33, 36, 37, 39, 43]
Apoptosis	*BIRC3, TNFRSF5, TRAF3, TRAF5, BTK, APAF-1, XPB*	[30, 36, 39]
Metabolism	*UBD, SYK, E2F5, SP140, PFTK1, LPXN, PTPRC, PTPN1, TNFRSF5, EIF4B, BTAF1, AMPD3, POU2AF1, EGR2, ENPP2, BTK, ICSBP1*	[30, 33]
Cell Cycle Control	*CDKN2A, CUL1, TP53, ARID3A, JUN, JUNB, JUND, FOS, EVI5, TMEM209, ZC3HC1*	[27, 28, 33, 36–39, 41, 43]
Lymphocyte Development and Regulation	*NOTCH2, NOTCH1, SPEN, DTX1, SWAP70, MAML2, BTK, CXCR4, ARID3A, KLF2, NOTCH3, NOTCH4, PAX5, MAP3K8, IRF5*	[27, 28, 33, 34, 36, 37, 39–43]
Chromosomal and Transcriptional Regulation	*MLL2, ARID1A, EP300, CREBBP, SIN3A, TBL1XR1, GPS2, SMYD1, MLL3, ARID4A, HIST1H1D, HIST1H1E, HIST1H2BI, HIST1H4H, SMARCA2, CHD2, BCOR, CBFA2T3, BCL6, POT1, ILF1*	[27, 28, 33, 34, 36–38, 40]

While each study puts forth potential genetic targets for unraveling the pathogenesis of SMZL, there are still wide discrepancies among which targets will be most fruitful for further investigations. The chromosomal and genetic abnormalities most commonly annotated, along with their frequencies seen across SMZL, are provided in Table 2. Insight into the abnormal genetic landscape and transcriptional regulation of SMZL has presented a plethora of candidates for diagnosing and elucidating SMZL, but similar to the results of cytogenetic studies, conclusions identified from DNA-level mutations do not provide transparent explanations for fully deciphering the biology behind the progression and heterogeneity of SMZL.

**Table 2:** Molecular aberrations most prevalent in SMZL, and the frequency of occurrence in SMZL cases

GENETIC ABNORMALITIES		
***Gene***	***Mutation frequency***	***Citation***
*NOTCH2*	∼ 40%	[27, 28, 33, 34, 36, 37, 39, 40, 44]
*KLF2*	20% - 40%	[28, 41, 42]
NF-κB pathway *(CARD11, IKBKB, TNFAIP3, TRAF3, BIRC3, etc.)*	35% - 45%	[28, 29, 31, 33, 35–37, 41]
*MYD88*	10% - 15%	[28, 33–35, 41]
*TP53*	10% - 20%	[28, 37, 41, 45–47]
**CHROMOSOMAL ABERRATIONS**		
***Location***	***Incidence***	***Citation***
7q Deletion	30% - 40%	[10, 16, 19–27, 43, 48–53]
3q Gain	10% - 20%	
Misc. Gains (6p, 8q, 9q, 12q, 18q)	8% - 18%	
Misc. Losses (6q, 8p, 14q, 17p)	8% - 16%	

To better understand SMZL, studies into various regulatory mechanisms should be conducted as an attempt at explaining the heterogeneity among chromosomal and genetic aberrations. One mechanism of post-transcriptional regulation is via epigenetic modifications, primarily being methylation of DNA promotor sequences. Deregulated DNA methylation has been implicated in the development of similar B-cell malignancies [54–58], and hence, Arribas *et al*. published a genome-wide DNA-promoter methylation profiling study in an effort to characterize the differential patterns within SMZL [59]. A cohort of patients was identified with significantly increased promotor methylation, and it was associated with a decreased OS compared to patients without the increased methylation profile. Additionally, certain promoter methylation patterns were identified and shown to affect the same biological pathways that were implicated in the genetic studies. While methylation patterns provide one option for mechanistic regulation studies, there are other molecular components worthy of investigation as well in an effort to improve comprehension of SMZL pathology.

### MicroRNAs and B-cell lymphomas

Non-coding RNA molecules are regulatory biological elements that warrant further investigation due to their well-established mechanistic impacts and their relationships with genetic and chromosomal aberrations. MicroRNAs (miRNAs), a type of non-coding RNA, are 20 to 22 nucleotide post-transcriptional regulators that have been heavily researched and reported on for their role in various cancers over the past decade [60–62]. MicroRNAs function by targeting complementary messenger RNAs (mRNAs), allowing them to regulate almost any cellular process that is a result of translation. The idea that miRNAs could play a role in lymphomagenesis originated from evidence that the miR-15/16 cluster was frequently deleted in chronic lymphocytic leukemia (CLL), resulting in the loss of tumor suppression [63]. Following this breakthrough, a plethora of studies were conducted to identify other miRNA in CLL pathogenesis as well as other lymphomas [64–66]. By exposing the oncogenic role of miRNA, they became an option for new treatment targets [67], diagnostic and prognostic markers [68, 69], and mechanism manipulation candidates [68, 70].

Preliminary studies have been conducted on the miRNA profile of SMZL, but the role for specific miRNA on SMZL pathogenesis remains to be discovered. Previous investigations into miRNA signatures of similar neoplasms may be informative for deciphering mechanistic impacts of deregulated miRNA in SMZL. Fortunately, unlike SMZL, many B-cell lymphomas have had their miRNA profiles investigated and reviewed extensively. Some miRNAs that are recurrently deregulated among B-cell lymphomas have been elucidated due to their role in B-cell development, migration, or activation [71]. Additional miRNAs also repeatedly identified in B-cell lymphomas are deregulators of “hallmark” cancer functions such as increased proliferation, evasion of suppressors, mortality resistance, and others [72]. The miRNAs consistently identified across B-cell lymphoma studies to be abnormally expressed include: miR-34a, miR-155, the miR-17/92 cluster, miR-21, and miR-150, with many more additional markers also discussed [62, 68, 71–74]. Many of these markers have also been found to be differentially expressed in SMZL miRNA profiles, but due to the exhaustive reporting on these miRNAs in B-cell lymphomas, no further discussion will be provided in this review regarding their potential role in lymphomagenesis. Instead, this review will provide an overview of the unique miRNAs hypothesized to play a role in SMZL pathogenesis. Furthermore, we propose why these miRNA targets warrant future investigations and discuss their oncogenic potential. A summary of all miRNAs reported to be relevant to the biology of SMZL is captured in Table 3.

**Table 3:** Characterization of the miRNA expression (and their location) in SMZL, inclusive of all previously published, relevant studies

miRNA	Location	SMZL expression status	Citation
miR-155	21q21.3	O	[77–79]
miR-451	17q11.2	O, OL	[73, 77]
miR-486	8p11.21	O, OL	[73, 77]
miR-146a	5q33.3	O, OL	[79, 80]
miR-494	14q32.2	O	[79]
miR-34a	1p36.22	O	[78, 79]
miR-193b	16p13.12	O	[78]
miR-100	11q24.1	O	[78]
miR-330	19q13.32	O	[78]
miR-21	17q23.1	O, UL	[77–79]
miR-144	17q11.2	OL	[73]
miR-204	9q21.12	OL	[73]
miR-212	17p13.3	OL	[73]
miR-409-3p	14q32.31	OL	[73]
miR-421	Xq13.2	OL	[73]
miR-432	14q32.2	OL	[73]
miR-487a/487b cluster	14q32.31	OL	[73]
miR-520d	19q13.42	OL	[73]
miR-542-3p	Xq26.3	OL	[73]
miR-574	4p14	OL	[73]
miR-595	7q36.3	OL	[73]
miR-650	22q11.22	OL	[73]
miR-29a/29b-1 cluster	7q32.3	U	[75–77]
miR-127	14q32.2	U, OL	[73, 77, 78]
miR-139	11q13.4	U, OL	[73, 77]
miR-335	7q32.2	U	[21, 76, 77]
miR-411	14q32.31	U	[77]
miR-593	7q32.1	U	[21, 76]
miR-129-1	7q32.1	U	[21, 76]
miR-139-5p	11q13.4	U	[79]
miR-345	14q32.2	U	[79]
miR-95	4p16.1	U, OL	[73, 79]
miR-138	3p21.32	U	[79]
miR-125a-5p	19q13.41	U	[79]
miR-126	9q34.3	U, OL	[73, 79]
miR-146b-5p	10q24.32	U	[79]
miR-223	Xq12	U	[80]
miR-377	14q32.31	U	[78]
miR-27b	9q22.32	U	[78]
miR-145	5q32	U	[78]
miR-376a/376b cluster	14q32.31	U	[78]
miR-381	14q32.31	U	[78]
miR-494	14q32.31	U	[78]
miR-382	14q32.31	U	[78]
miR-154	14q32.31	U	[78]
miR-410	14q32.31	U	[78]
miR-758	14q32.31	U	[78]
miR-485-3p	14q32.31	U	[78]
miR-136	14q32.31	U, OL	[73, 78]
miR-379	14q32.31	U	[78]
miR-338	17q25.3	U	[78]
miR-107	10q23.31	U	[78]
miR-24	9q22.32	U	[78]
miR-328	16q22.1	U	[78]
miR-199a	19p13.2	U	[78]
miR-483	11p15.5	U	[78]
miR-589	7p22.1	U, UL	[78]
miR-182/96/183 cluster	7q32.2	UL	[21, 76]
miR-26b	2q35	UL	[78]
miR-19b	13q31.3	UL	[78]
miR-660	Xp11.22	UL	[78]
miR-448	Xq23	UL	[78]
miR-646	20q12.33	UL	[78]
miR-323	14q32.31	UL	[78]
miR-567	3q13.2	UL	[78]
miR-141	12p13.31	UL	[73]
miR-199b	9q34.11	UL	[73]
miR-200c	12p13.31	UL	[73]
miR-210	11p15.5	UL	[73]
miR-663	20p11.1	UL	[73]
miR-99a	21q21.1	UL	[73]

Abbreviations: O, overexpressed compared to normal spleen; U, under-expressed compared to normal spleen; UL, underexpressed compared to B-cell lymphomas; OL, over-expressed compared to B-cell lymphomas.

### miRNAs in SMZL

The first study to propose a biological effect by miRNA on SMZL was published by Ruiz-Ballesteros *et al*. in 2007 and reported decreased expression levels of miR-29a and miR-29b-1 [75]. The two miRNA were chosen for the first study due to their proximity to the commonly deleted 7q region mentioned above, and miR-29a is also known to have the potential to target and regulate *TCL1A*, a predicted oncogene in SMZL [30, 81]. Subsequent studies followed, also investigating the association between miRNA levels and 7q mutational status. Watkins *et al*. reported a reduction in the expression of 7 miRNA located at 7q32, consistent with the chromosomal report of LOH at that location [21, 76]. This sentinel finding ignited curiosity within the scientific community regarding the role of miRNA and 7q LOH and should be further investigated to identify possible mechanistic connections.

The miRNA landscape of SMZL was also studied beyond the miRNA located at 7q32. Bouteloup *et al*. identified a significant variation in expression of 7 miRNAs when comparing healthy samples to SMZL samples, with two of the identified miRNA being located at 7q [77]. Additionally, miR-21 over-expression was associated with the aggressiveness of SMZL cases in their study. A report published in 2012 investigated 8 different B-cell lymphomas for specific miRNA signatures within each respective malignancy [73]. SMZL was included in this study, finding 26 different miRNAs to be differentially expressed in SMZL, with 20 being upregulated and 6 being downregulated in SMZL when compared to the other B-cell lymphomas. Arribas *et al*. conducted a more comprehensive analysis of the miRNA profile for SMZL, finding over 30 miRNAs differentially expressed when compared to reactive spleens, with 9 miRNAs differentially expressed from similar B-cell lymphoma miRNA profiles [78]. Lastly, a study designed to investigate the SMZL miRNA profile, as well as the role of Hepatitis-C Virus on SMZL miRNA, determined a key role for 12 differentially expressed miRNAs in SMZL [79].

Incredibly, across each of the studies, very few miRNAs were found to be differentially expressed in multiple instances (Table 3). The variability among the miRNA profiles once again demonstrates the heterogeneity across SMZL, but due to the preliminary nature of the SMZL miRNA knowledge, as well as the vast regulatory ability of each miRNA, further investigation into their role on SMZL pathogenesis holds many opportunities. The identification of potential candidates for mechanistic studies is the next step to uncovering how post-transcriptional regulation influences SMZL progression. Taken together, the following miRNA are proposed candidates for subsequent studies that should be conducted in an effort to elucidate the biology of SMZL.

### Mechanistic impact of miRNA

The nearly infinite number of mechanistic roles performed by miRNA have been well established in the literature. Sifting through the abundance of the miRNAs for their role in diseases, however, can prove tedious. Hence, we took a reductionist approach by looking at the known role of miRNA in SMZL that are located within the 7q region and a select few of the miRNA that target *caveolin-1* (*CAV1*). We listed each of the miRNA identified to be differentially expressed in SMZL signatures (Table 3), and we propose 7 of those miRNAs contain potential to be significant contributors to SMZL pathogenesis. These miRNAs are categorized into two different designations in an attempt to delineate the roles that these pathways may play on this specific lymphomagenesis (Table 4).

**Table 4:** Identification of the miRNA at the 7q region, or those that target the oncogene/tumor suppressor caveolin-1

Category	miRNA	Regulation type	Proposed SMZL regulation target
Transcribed at 7q	miR-29a/b-1 cluster	Tumor Suppressor	*TCL1A*
	miR-129-1	Tumor Suppressor	*BCL2*
	miR-182/96/183 cluster	Tumor Suppressor or Oncogene	*FOXO1*
	miR-335	Tumor Suppressor	Rb1, *BCL-w*
Target CAV1	miR-199a	Tumor Suppressor	*IKKβ, CAV1*
	miR-376	Tumor Suppressor	*IGF1R, CAV1/IGF1R/SRC*
	miR-485	Tumor Suppressor	*CAV1*

The proposed target of regulation within SMZL cells is also described.

The miRNAs located at the 7q region have been hypothesized to play a role in SMZL progression, and due to the frequency of 7q chromosomal abnormalities seen in SMZL, should be investigated for their role. Hence, the first categorical designation will be miRNA that are transcribed within the 7q region. This includes miR-29a/b-1, miR-129-1, the miR-183/96/182 polycistron, and miR-335.

The other category corresponds to miRNA that directly target the mRNA of *CAV1*, a gene located at 7q31 that has been implicated in similar B-cell lymphomas. There were 9 miRNAs differentially expressed in previous studies that target *CAV1*, and of the 9, this review will discuss 3 of the miRNAs that could be crucial for exposing a role for *CAV1* in SMZL pathogenesis. These miRNAs discussed below include: miR-199a, miR-376, and miR-485. The possibilities for the role of miRNAs in SMZL are almost limitless, but this review provides an evidence-based list of 7 miRNAs that could be crucial to understanding this malignancy.

### miRNA located at 7q

The LOH near the 7q32 chromosomal region is a disruption unique to few lymphomas, and the loss seen in up to 40% of SMZL patients is the highest among B-cell lymphomas. As previously discussed, this unique aberration is being utilized in diagnostic techniques for SMZL, but the biological mechanisms behind these losses are difficult to identify. While some groups have hypothesized that miRNA could play a role in this LOH, and others have even looked at the miRNA expression profiles within the 7q32 region in SMZL tissues, the mechanistic role for these miRNA remains to be discovered. The 4 miRNAs below demonstrated significantly lower expression in SMZL cases against healthy samples or similar malignancies. This differential expression was seen across multiple studies in most cases, and the variable expression's consequent downstream regulatory alterations may prove important for SMZL pathogenesis.

### miR-29a/b-1 cluster

The miR-29 family contains a two-member miRNA cluster located distally to a commonly deleted region of 7q, 7q32. The miRNA family contains four members, miR29a, miR-29b-1, miR-29b-2, and miR-29c. While the cluster contains the same seed sequence, resulting in many of the same targets and regulations, only miR-29a and miR-29b-1 are located on 7q. miR-29 is commonly under-expressed in SMZL spleens when compared with healthy tissues. This decrease of expression is consistent with the expression seen in similar B-cell neoplasms, CLL and mantle cell lymphoma (MCL), as well as many other malignancies [61, 67, 82–85]. The variable expression of miR-29 has been viewed with such importance that utilization in diagnostic, and more importantly prognostic, lymphoma designations have been proposed [86]. The regulatory role of miR-29 has been implicated in immune regulation, cell proliferation, differentiation and apoptosis pathways, metastatic interference, and even epigenetic modulation [87]. Due to the myriad of regulatory mechanisms and variable malignancies affected, miR-29 acts as a tumor suppressor in some situations while displaying oncogenic tendencies in others. The replicated under-expression of miR-29 seen in SMZL is consistent with miR-29 levels observed in similar B-cell malignancies, indicating that miR-29 primarily is acting as a tumor suppressor in SMZL. Further, in situations of LOH at or near the 7q32 region, there could be complete loss of miR-29 transcripts resulting in possible loss of its tumor suppressive activity and a much more aggressive progression of the malignancy. Further studies are necessary to find the precise mechanistic role of 7q32 status on miR-29 and the overall impacts on SMZL progression.

Due to the established research into the role of miR-29 in lymphomas similar to SMZL, the hypothesized mechanistic investigations have promising directionality. A primary target of miR-29a is the mRNA of *TCL1A*, and upon binding to the *TCL1A* mRNA it deactivates the oncogenic properties of the impending protein. *TCL1A* is an oncogene that has been shown to enhance cancer progression via its role in cell survival pathways, and it is commonly over-expressed in aggressive subtypes of many cancers. It has been shown that miR-29a acts as an inhibiting regulator of *TCL1A* in CLL [88], and as miR-29 has a reduction in expression, *TCL1A* demonstrated the corresponding increase in expression in those cases. *TCL1A* has been shown to have increased expression among SMZL cases [30], and thus, a similar mechanistic role for miR-29 in SMZL is not out of the question. As mentioned above, in SMZL cases with which the 7q32 region is deleted, miR-29a could also be deleted. It has also been observed in former studies that miR-29a may be under-expressed in SMZL cases, regardless of 7q mutational status [75]. This deletion or reduction in expression could result in a lack of *TCL1A* silencing, and consequently, would promote lymphomagenesis due to the down-stream protein's increased activation and corresponding effects. Due to the multifaceted regulatory mechanisms of miR-29, multiple cellular processes are disrupted upon its down regulation, as is seen in SMZL, but specific investigations into the tumor suppressive mechanisms, like the impact on *TCL1A*, are necessary for the exploitation of miR-29 in treatment and diagnostic opportunities.

### miR-129-1

The miR-129 family is composed of miR-129-1 and miR-129-2, with the former being located at 7q32 within the region most commonly deleted in SMZL presentations. The two miRNA have almost identical seed sequences, resulting in similar functionalities. miR-129 has been primarily identified for its tumor suppressive role in various tumorigeneses, but oncogenic properties of the miRNA have been discovered as well. miR-129-1 was shown to have repressed expression of in SMZL. Thus, tumor suppression is most likely the route of regulation for this specific malady. Other cancers have also been found to display under-expression of miR-129, with a plethora of proposed mechanistic explanations from cell proliferation, cell cycle, apoptotic, metastatic, and autophagy pathway regulations [89]. Karaayvaz *et al*. investigated the regulation by miR-129 as a tumor suppressor, finding that through direct targeting of *B-cell lymphoma 2* (*BCL2*), miR-129 induces apoptotic mechanisms and inhibits cell proliferation via cell-cycle arrest in colorectal cancer cells [90]. Consequently, upon decreased expression of miR-129, each of the tumor suppressive mechanisms are also stifled. A similar situation may occur in SMZL, as miR-129 shows decreased expression compared with healthy samples. Further, *BCL2* has been found to be over-expressed in SMZL signatures [78], providing additional evidence for the practicality of the miR-129 regulatory impact. Investigations into the role of miR-129-1 on SMZL pathogenesis and 7q LOH may prove beneficial based on reported information mentioned above, and thus, the mechanistic understanding of miR-129 related to SMZL should be improved.

### miR-182/96/183 cluster

The miRNA polycistron consisting of miR-182, miR-96, and miR-183 is considered a cluster due to the miRNAs’ proximity to one another. The miR-183 cluster is located at 7q32, and the miRNAs possess similar seed sequences, resulting in many shared functionalities. These miRNAs are typically seen over-expressed in various cancers, but under-expression has also been observed, demonstrating the dual tumor-suppressive and oncogenic role of the polycistron. More specifically, an increased expression of miR-183 was reported in lung cancer tissues [91], but in a separate study, miR-183 was found to have decreased expression in the peripheral blood of lung cancer cases [92], indicating miR-183 is utilized for contradictory mechanistic interactions in hematological and tissue malignancies. As for previously published miRNA data in SMZL, the miR-183 cluster has been shown to be under-expressed when compared to similar B-cell lymphoma samples [76]. The expression values when compared to control samples were unavailable, though. Thus, the under-expression seen in SMZL could be the result of an increase in tumor suppressive properties, or it could be an indication for a reduction in oncogenic effects compared to the B-cell lymphoma counterparts. A polymorphic mutation in approximately one quarter of SMZL cases has also been found at the region containing miR-182, indicating possible loss of function in some SMZL cases [76], but it has also been shown that the polymorphism can be found among healthy populations at an only slightly lower occurrence bringing into question its carcinogenicity [93]. This alteration in miR-182 expression should first be investigated for the in-question dependence to the aforementioned polymorphism, but regardless, the under-expression of the entire miR-183 cluster seen in SMZL indicate it should be assessed for its possible mechanistic role in the 7q deletion.

A variety of malignancies have been investigated for connections to the aberrant role of miR-183 cluster members on carcinogenesis, but conflicting mechanistic actions in varying tissues and malignancies make this evaluation inconsistent. The cluster is commonly over-expressed in many solid tumors, but there has also been significant under-expression of the cluster in other malignancies [94]. Even among hematological malignancies expression is variable. MCL cases present with an increased miR-182 expression [95], but CLL cases showed a p53 regulated decrease in miR-182 expression [73, 96]. Mechanistic relationships between miR-183 cluster members have stretched to many pathways and functions. There has been regulation in cell proliferation, cell apoptosis, cell migration, immune signaling, and DNA repair mechanisms, indicating the wide carcinogenic outreach of the cluster [97]. Connections to sonic hedgehog signaling pathways, Pro-apoptotic Programmed Cell Death (PDCD) family regulation, and regulation of Forkhead Box O (FOXO) subfamily signaling, just to name a few, have been published for explanations of the role the miR-183 cluster, or one of its’ members, has on various tumorigenesis processes [95, 98–109]. With such an impactful list of vetted carcinogenic targets, studies establishing the miR-183 cluster expression profile in SMZL against healthy/control tissues, and further investigation into the role of the cluster on SMZL pathogenesis, could renders vital for understanding the possible function of 7q LOH in SMZL overall progression.

### miR-335

The final miRNA of interest located within the 7q region is miR-335. Similar to the other miRNA of interest, miR-335 was significantly under expressed in SMZL samples when compared to healthy controls. Research on miR-335 has been done primarily on solid tumor malignancies but the mechanistic conclusions in studies have the potential to be translated further [110]. miR-335 has been discussed for its role as an oncogene, but it has contrarily been found to act as a tumor suppressor in other malignancies. Many of the cancers displaying an under-expression of miR-335, like SMZL, point to tumor suppressive mechanisms as its role in carcinogenesis.

One mechanism of action with far-reaching impacts demonstrated the role of miR-335 in p53 regulation. Scarola *et al*. found that miR-335 targets and represses *retinoblastoma 1* (*RB1*) resulting in the up-regulation of p53 [111], but p53 pathway activation results in further up-regulation of miR-335, hence triggering a positive feedback loop. Thus, in a malignancy like SMZL when miR-335 is under-expressed, or in a case of 7q deletion possibly eliminating the coding regions for miR-335 rendering it almost nonexistent, there is no down regulation of *RB1* and no downstream activation of p53, resulting in a huge decrease in the wide-spread tumor suppression. Additionally, *TP53* deletions and mutations have been shown in 15% - 25% of SMZL cases [27, 28, 38, 47], which would also result in disruption of the aforementioned positive feedback loop. Hence, this would provide an additional explanation for the decrease in miR-335 expression. Further investigations into this mechanistic relationship may prove crucial for understanding miR-335 as well as *TP53* functioning in SMZL.

In other work, miR-335 has been shown to regulate BCL-w, a member of the BCL-2 protein family, suppressing its role in cell proliferation pathways, resulting in apoptosis of tumor cells [112, 113]. Again, as miR-335 is acting as a tumor suppressor, its under-expression would result in less suppression of *BCL-W* allowing the tumor cells to proliferate much easier promoting their survival. Either of the aforementioned mechanisms could prove helpful in understanding SMZL, as p53 is a universal tumor suppressor that impacts all malignancies, and the B-cell lymphoma protein family is extremely homogeneous and miR-335 targeting of other *BCL* homologues should not be overlooked.

### miRNA targeting *CAV1* in SMZL

The LOH at 7q in SMZL has not been fully elucidated due to the many regulatory mechanisms that are possibly responsible or work in conjunction to impose the aberration. The fragile site FRA7G overlaps with part of the 7q region commonly deleted in SMZL, and within that site, at 7q31.2 to be exact, the tumor suppressor and oncogene, *CAV1*, resides. Therein other instances of LOH involving *CAV1* resulted in malignant transformations [114, 115]. When *CAV1* is coupled with an oncogene, *CAV1* knockout mice appear more prone to progress to aggressive forms of cancers than in mice without the *CAV1* knockout [116]. This impact could prove crucial for deciphering SMZL within an *in vivo* model and hence has been recently proposed [117]. The fragility and importance of the 7q region in SMZL almost certainly implicates *CAV1* as a significant player in the disease etiology due to its crucial chromosomal location. Thus, the mechanisms and regulatory pathways extrapolating the role of *CAV1* in SMZL pathogenesis are worthy of further investigation.

**Figure 1:**
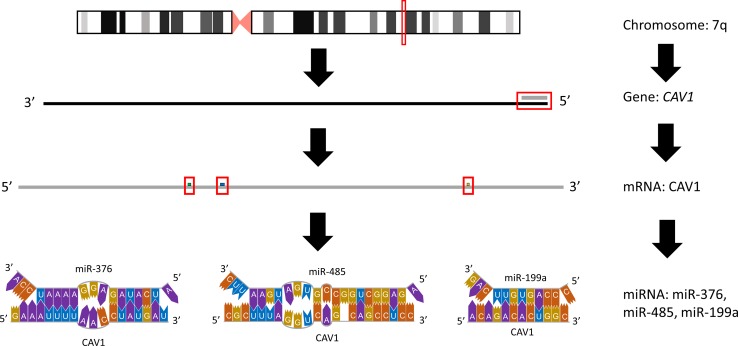
Graphical representation of affected region on 7q The predominantly affected region of chromosome 7 frequently mutated or lost in splenic marginal zone lymphoma is depicted, inclusive of the miRNA within this region that target *CAV1.*

There have been discrepancies among reported results regarding *CAV1* status in SMZL cases. Ruiz-Ballesteros *et al.* showed decreased expression of *CAV1* [30], but Watkins *et al.* found no differential regulation of *CAV1* in SMZL samples [21]. These inconsistencies may be a result of additional molecular regulators and their impacts. There are 9 miRNAs reported to be aberrantly expressed in the SMZL profile, that have the ability to target *CAV1*, and thus, it would be no surprise if miRNAs regulation on *CAV1* may be the culprit responsible for these discrepancies. Further, *CAV1* expression has been reported to be independent of 7q mutation status also supporting the idea that regulatory mechanisms beyond chromosomal loss are almost certainly at work. *CAV1* has been shown to be a crucial piece in immune functioning and dysregulation in malignancies similar to SMZL, and in order to decipher the role of *CAV1* on SMZL, in cases of 7q LOH or in cases with 7q intact, miRNA regulation should be investigated for their impact. There are 3 miRNAs (miR-199a, miR-376 cluster, and miR-485) that will be discussed next that present viable options for *CAV1* regulation and, in turn, impact SMZL pathogenesis (Figure 1).

### miR-199a

The decreased expression of miR-199a seen in SMZL is consistent with previously published literature. In studies to this point, miR-199a has demonstrated tumor suppressive properties, and hence, has been under-expressed in many of the malignancies being investigated. Due to its importance as a tumor suppressor it has been studied for its prognostic significance and potential as a treatment target in solid and hematological malignancies [118]. Many studies have demonstrated the multifaceted, tumor suppressive properties of miR-199a. The decreased expression of miR-199a induces an increase in IKKβ activity, further stimulating the NF-κB pathway, which results in an enhanced tumor environment and chemoresistance [119–121]. The proposed tumor suppressive mechanisms of miR-199a have also included downregulation of proto-oncogenes and their corresponding pathways [122], as well as regulation of mammalian target of rapamycin, cell migration, and apoptotic pathways [118]. In the malignancy most similar to SMZL, diffuse large B-cell lymphoma (DLBCL), the increased expression of miR-199a among patient cohorts has corresponded to a more favorable progression free survival and OS [118, 123].

While pathway regulation is important for elucidating the role of miR-199a, in order to exploit miR-199a for more treatment and prognostic options precise mechanistic understanding must be improved. There has been research into the mechanistic relationship that miR-199a and the tumor suppressor and oncogene *CAV1* may have. miR199a has been shown to directly target *CAV1* and, in turn, affect the resulting mechanisms in which *CAV1* may be involved [124]. Subsequent studies further supported this relationship and have found miR-199a significantly inhibits *CAV1* expression and function [125]. Thus, when miR-199a is shown to have decreased expression in SMZL cases, an increased expression of *CAV1* could be expected, which has shown to be detrimental in other cancers [126–130]. Experimental investigations into the relationship between *CAV1* and miR-199a may not only prove beneficial for understanding its role in SMZL progression but could render fruitful for deciphering other malignancies and diseases as well.

### miR-376 cluster

An additional target of *CAV1*, the miR-376 cluster, was also significantly under-expressed in SMZL compared to non-tumoral controls [78]. The miR-376 family has not been studied extensively, but it has been shown to act with oncogenic properties in some malignancies while displaying tumor suppressive functions in other cancers, resulting in proposed utilization as a biomarker [131–133]. The decrease in miR-376 expression in SMZL cases indicates tumor suppressive properties, but the mechanistic understanding of miR-376 remains unknown. miR-376 has been shown to impact cancer progression via cell cycle progression, cell migration and invasion, and autophagy [134–136]. One mechanism of tumor suppression is the direct regulation of *IGF1R* by miR-376a and miR-376c, resulting in decreased migration and proliferation [134]. Thus, upon reduction of miR-376a expression, as is seen in SMZL, *IGF1R* becomes fully activated, promoting tumor progression. Additionally, IGF1R forms a complex with CAV1 and SRC in order to induce anti-apoptotic mechanisms [137]. When inhibition of CAV1 or IGF1R was applied, it corrupted the complex allowing apoptotic molecules to resume mitigating tumor development. This identifies another route that miR-376 may serve as a tumor suppressor, as it would be able to directly target and suppress *CAV1*, *IGF1R*, or both, to disrupt their oncogenic mission. Hence, the decrease in miR-376 in SMZL would limit the capacity for these tumor suppressive functions.

Arribas *et al.* hypothesized additional predicted targets of miR-376 that have been shown to act in tumorigenesis: *CD44*, *MUM1*, *DLEU1*, *IL2RA*, *IL7*, *IRTA4*, and *FOXP1* [78], all of which could potentially be upregulated in SMZL. Upregulation of *FOXP1*, for example, has been demonstrated in SMZL [78]. It is similarly upregulated in DLBCL signatures, resulting in increased oncogenic activity [138]. This upregulation of *FOXP1* and subsequent increase in malignant activity would be consistent with a decrease in miR-376 expression, and thus, this relationship should be further explored. Finally, miR-376 has been shown to undergo RNA editing, resulting in altered mRNA targets, in germinal center based B-cell lymphomas [139]. This alteration in targets can result in a multitude of aberrant regulations, possibly even becoming carcinogenic. And, while the cellular origin of SMZL is still controversial, it's plausible that RNA editing within the miR-376 could assist in tracking etiology in a subset of cases. Each of the aforementioned mechanisms present the capability to contribute to SMZL pathogenesis understanding the impact of miR-376 on SMZL and other malignancies should be pursued.

### miR-485

The final miRNA of interest in this review is miR-485. Arribas *et al*. demonstrated significant under expression of miR-485 in SMZL samples [78]. The decreased expression is consistent with many of the previously published findings on miR-485, and it indicates the miRNA exhibits primarily tumor suppressive properties on SMZL pathogenesis. Increased expression of miR-485 has corresponded to enhanced treatment resistances in tumor cells [140, 141], and increased expression has also been shown to impact cell migration and invasion, colony formation, cell viability, and mitochondrial functioning in tumor cells, resulting in decreased cell viability [142, 143]. Additionally, decreased expression of miR-485 has corresponded to less desirable outcomes in clinical studies indicating possible functionality as a biomarker [144, 145]. One proposed mechanism of the tumor suppressive role of miR-485 is the result of a single-nucleotide polymorphism (SNP) at the miR-485 binding site [146]. This SNP abrogates the ability of the miRNA to accurately bind to target mRNAs resulting in loss of tumor suppressive function. Chen *et al*. also proposed that miR-485 targets pathways involved in topoisomerase inhibition, and through decreased expression of miR-485, there is decreased sensitivity in related treatments [140]. The role of miR-485 on SMZL pathogenesis has yet to be investigated, but its interaction with *CAV1*, a predicted target of miR-485, is worthy of a further look. The previously proposed mechanisms from altered miRNA regulation due to SNPs or direct regulation of *CAV1* resulting in downstream effects are both viable possibilities and could be investigated in SMZL cellular environments.

## CONCLUSIONS

Splenic marginal zone lymphoma is an indolent, non-Hodgkin lymphoma with an OS of over 10 years in most cases. Approximately a third of these cases become aggressive and possibly transform to a much more lethal lymphoma, cutting the OS almost in half for those patients. Mechanistic understanding behind this unfavorable prognosis remain unknown, despite a plethora of chromosomal and genetic investigations. The lack of cohesive results in much of the literature may be the result of additional regulatory mechanisms, resulting in abnormal functioning and interactions at the chromosomal and genetic levels. The role of miRNA in cancer is a growing investigative interest, as elucidation of their regulation has proven enlightening for deciphering various malignancies and their progression. This review discusses the discrepancies among current data regarding understanding of SMZL pathogenesis and proposes miRNA regulation to be a possible culprit. The regulation by 7 miRNAs previously identified to have altered expression in SMZL are discussed, and possible mechanisms for their impact on SMZL progression are proposed based on previous findings in other malignancies. The miRNA located within the most commonly effected chromosomal region in SMZL, 7q and the miRNA that target *CAV1*, a gene implicated in many cancers and located at 7q31, were also a focus of this review. Further investigations into the mechanistic role of miRNA in SMZL may provide insight into the disease etiology and could identify possible candidates for prognostic biomarkers and treatment targets, improving acumen for this disease entity.
